# Body Mass Index Trajectory and Incident Mild Cognitive Impairment Among African American Older Adults

**DOI:** 10.1093/geroni/igab046.436

**Published:** 2021-12-17

**Authors:** Adrienne Aiken Morgan, Ana Capuano, Robert Wilson, Lisa Barnes

**Affiliations:** 1 University of North Carolina at Chapel Hill, North Carolina, United States; 2 Rush, Chicago, Illinois, United States; 3 Rush University Medical Center, Chicago, Illinois, United States

## Abstract

Previous research suggests a decline in body mass index (BMI) among older adults is associated with negative health outcomes, including mild cognitive impairment (MCI) and incident dementia (Gao et al., 2011). However, few studies have examined BMI longitudinal trajectories and how they change after MCI diagnosis among older African Americans. To characterize trajectories of change in BMI among older African American participants with no cognitive impairment at baseline we used data from the Minority Aging Research Study, MARS (N=408, 76.5% women, mean age = 73.5, mean education = 15.0). We constructed piecewise linear mixed-effects models that included a random intercept and two random slopes. The first slope began at baseline. The second slope began at MCI diagnosis allowing for acceleration in the rate of decline after the diagnosis. The results showed BMI declined over time (B=-0.19, SE=0.04, p<.001), and there was a faster decline after MCI (additional decline, B=-0.18, SE=0.068, p=.007). In a second model controlling for age, higher education was associated with a lower BMI at baseline (B=-0.36, SE=0.092, p<.001) but slower decline before MCI (B=0.02, SE=0.006, p=.001). However, after MCI the decline of participants with higher education was faster (B=-0.06, SE=0.022, p=.003). These results suggest an accelerated decline in BMI following MCI diagnosis, with higher education related to an even faster BMI decline, possibly a consequence of cognitive reserve.

